# Targeted Drug Delivery in the Suprachoroidal Space by Swollen Hydrogel Pushing

**DOI:** 10.1167/iovs.17-23758

**Published:** 2018-04

**Authors:** Jae Hwan Jung, Patcharin Desit, Mark R. Prausnitz

**Affiliations:** 1 School of Chemical and Biomolecular Engineering, Georgia Institute of Technology, Atlanta, Georgia, United States; 2 Department of Chemistry, Faculty of Science, Chulalongkorn University, Bangkok, Thailand

**Keywords:** hyaluronic acid, hydrogel swelling, microneedle injection, New Zealand white rabbit, ocular drug delivery, posterior segment, suprachoroidal space injection

## Abstract

**Purpose:**

The purpose is to target model drug particles to the posterior region of the suprachoroidal space (SCS) of the eye controlled via pushing by hydrogel swelling.

**Methods:**

A particle formulation containing 1% hyaluronic acid (HA) with fluorescent polymer particles and a hydrogel formulation containing 4% HA were introduced in a single syringe as two layers without mixing, and injected sequentially into the SCS of the rabbit eye ex vivo and in vivo using a microneedle. Distribution of particles in the eye was determined by microscopy.

**Results:**

During injection, the particle formulation was pushed toward the middle of the SCS by the viscous hydrogel formulation, but less than 12% of particles reached the posterior SCS. After injection, the particle formulation was pushed further toward the macula and optic nerve in the posterior SCS by hydrogel swelling and spreading. Heating the eye to 37°C, or injecting in vivo decreased viscosity and mechanical strength of the hydrogel, thereby allowing it to swell and flow further in the SCS. A high salt concentration (9% NaCl) in the hydrogel formulation further increased hydrogel swelling due to osmotic flow into the hydrogel. In this way, up to 76% of particles were delivered to the posterior SCS from an injection made near the limbus.

**Conclusions:**

This study shows that model drug particles can be targeted to the posterior SCS by HA hydrogel swelling and pushing without particle functionalization or administering external driving forces.

Worldwide, 285 million people have a visual impairment, and this number increases by approximately 7 million each year.^1,2^ Although many ophthalmic pharmaceuticals have been developed, people still suffer from diverse forms of ocular disease, particularly posterior-segment disease, such as age-related macular degeneration (AMD), diabetic macular edema (DME), and posterior uveitis, in part due to low drug bioavailability resulting from anatomic and physiologic ocular barriers.^3–5^

Intravitreal injection is widely used to treat posterior-segment disease; however, intravitreal injection is invasive, carries the potential for infection, and the vitreous humor is not the site of action for posterior-segment disease therapies.^2,6–8^ Targeting drug delivery to the chorioretinal layer, especially around the macula, where most posterior-segment disease is found, is expected to improve drug bioavailability and efficacy with reduced side effects.

Targeting the chorioretina can be achieved by drug injection into the suprachoroidal space (SCS), a potential space lying between the choroid and sclera.^2,9,10^ Following injection into the SCS, drug formulations flow circumferentially within the SCS from a typically anterior site of injection toward the macula and posterior pole. SCS injection (specifically, using a microneedle) can increase the bioavailability of drugs at their site of action, in particular in the chorioretinal layer to treat posterior-segment diseases (e.g., AMD, DME, and posterior uveitis), in a minimally invasive manner.^11–15^

Microneedles are tiny hypodermic needles typically measuring less than 1 mm in length that just cross the sclera and thereby target the SCS.^12–14,16^ Using a microneedle, drug formulations can be delivered directly into adjacent chorioretinal layers via SCS injection without needing to penetrate additional ocular layers (e.g., sclera, conjunctiva, and cornea). While scleral thickness varies among different people and locations on the eye, injection specifically at the sclera-choroid interface is facilitated by the feeling of a large resistance to injection into sclera compared to the relatively little pressure needed to inject in the SCS.^14^

The safety and tolerability of SCS injection using a microneedle have been shown in clinical trials.^17–20^ However, drugs injected into the SCS are delivered to multiple adjacent tissues: not only the posterior segment around the macula, but also the ciliary body and posterior segment tissues anterior to the macula.^21–23^ Therefore, improved SCS injection that targets specific areas within the SCS (e.g., adjacent to the macula) should increase drug efficacy and reduce side effects.^18,20–22^

In our previous study, we developed a micrometer-sized particle emulsion to control the location of drugs within the SCS. Due to their high density, the emulsion droplets could move under the effects of gravity and, by positioning the head appropriately, could direct drug delivery within the SCS, for example, toward the posterior pole.^23^ In another study seeking to localize delivery to the anterior SCS adjacent to the ciliary body, we developed a highly viscous drug formulation consisting of carboxymethyl cellulose for injection into the SCS. Due to its viscosity, the drug formulation stayed near the injection site without spreading posteriorly.^24^ In a final study, we used iontophoresis applied to control the particle localization within the SCS. By using drug particles carrying a charge, the drug particles could be directed to the posterior or anterior parts of the SCS based on electric field polarity.^25^

Hydrogels are used frequently in drug formulations because they are safe and often straightforward to modify.^26–29^ Hyaluronic acid (HA) hydrogels have been used widely for sustained ocular drug delivery, because HA not only is biodegradable and safe, but is a natural component of vitreous humor.^29–31^ HA hydrogels can by synthesized by chemical reaction with covalent bonding^32–34^ or can form physically at high HA concentration by hydrogen bonding between HA chains.^31,35^ Because the noncovalently bound hydrogel can be disrupted by shearing force due to the relatively weak hydrogen bond, this property facilitates injection at low viscosity during high shear in a small-bore needle^24^ and high mechanical strength and viscoelasticity once delivered when shear forces are low.^36–38^ In addition, hydrogels based on hydrogen bonding can disassemble upon dilution and spread within several hours in vivo by water influx due to osmotic pressure and temperature increase in the body.^27,31,39^

We proposed to use hydrogel swelling as a means to push drug particles through the SCS toward the posterior pole. Specifically, a solution of drug particles is first injected into the SCS, after which a swellable hydrogel is injected posterior to the limbus. Upon swelling, the hydrogel expands in volume and thereby pushes the drug particles away from the site of injection toward the posterior pole.

In this study, a physically synthesized HA hydrogel that is injectable and biodegradable was used to target drug particles to the back of the eye via the SCS. We hypothesized that drug particles injected to the SCS initially can be pushed by the HA hydrogel injection, due to the viscoelastic properties and mechanical strength of the hydrogel. After injection, the hydrogel can spread and swell in vivo within several hours due to water influx from surrounding tissues and temperature increase from room to body temperature. Since the spreading and swelling reduce the viscosity and mechanical strength of the hydrogel, the swollen hydrogel can flow from the anterior site of injection to the posterior SCS.^40,41^ The hydrogel flow can push and localize the drug particles further to the back of the eye around the macula and optic nerve. To our knowledge, this is first study using a hydrogel as a pushing material instead of a drug carrier in ocular drug delivery.

## Methods

### Experimental Design

**Figure 1:**
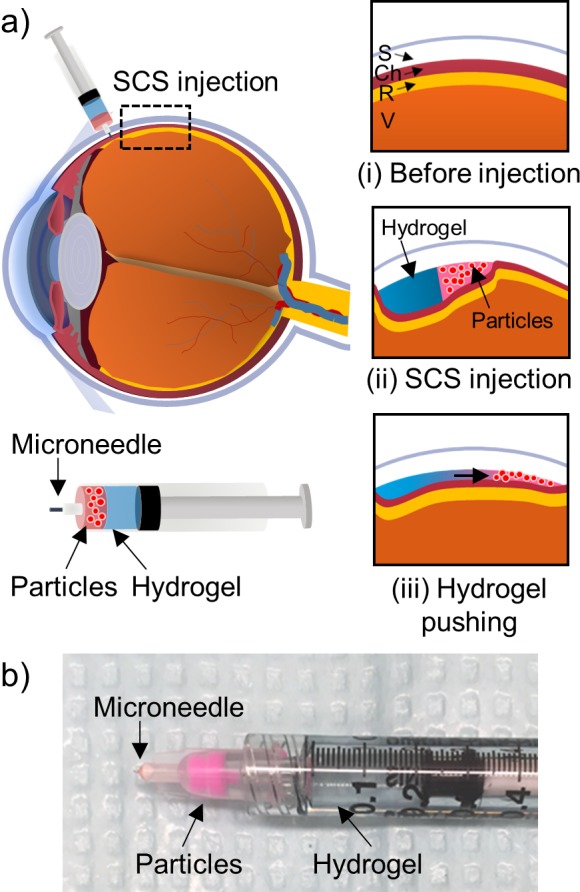
Drug delivery targeted by hydrogel pushing in the SCS of the eye. (a) Scheme illustration of targeted drug particle delivery by hydrogel pushing within the SCS. The injection is targeted to the SCS between the sclera and choroid (i), where a drug particle formulation is injected first, followed by a hydrogel formulation that pushes the particle formulation deeper into the SCS (ii). After injection, hydrogel swelling and flow within the SCS can push the particle formulation further toward the back of the eye (iii). (b) An assembled microneedle syringe containing the drug particle and pushing hydrogel formulations without mixing.

The first step in this study was to design a method for SCS injection that used the swelling of HA hydrogel to push a microparticle formulation to the posterior region of the SCS near the macula and optic nerve (Fig. 1a). We designed a first formulation to contain 2 μm diameter polymer microparticles as a model for drug particles, and a second formulation to contain HA hydrogel that can swell in the SCS after injection. The second formulation needed to have a viscosity greater than the first formulation so that it would push, rather than mix with the first formulation.^42–45^ Viscosity was controlled by varying HA concentration, as discussed below.

A final design consideration was that we wanted these two formulations to be stored and injected in a single syringe. Increased viscosity was used to limit mixing of the two formulations during storage. We theoretically estimated the specific viscosity of HA hydrogel depending on HA concentrations (see Supplemental Information, SI).^42,46,47^ Briefly, increasing HA concentration increased the specific viscosity of the hydrogel exponentially, which is believed to be due to gelation caused by crowding HA chains in solution with more interaction.

More specifically, the particle formulation was loaded in the front of the syringe, and the hydrogel formulation was placed behind. In this way, upon injection into the SCS, the particle formulation would enter first and the hydrogel formulation would follow, thereby pushing the particle formulation further into the SCS (Fig. 1a[ii]). Additional pushing by the hydrogel formulation can occur as the hydrogel swells and flows within the SCS after injection. At room temperature, the hydrogel behaves like a solid gel; however, at body temperature, the hydrogel can flow like a viscous liquid^27,31,39^ and thereby further push the particle formulation (Fig. 1a[iii]). The hydrogel also can swell due to diffusion and osmotic flow of water into the hydrogel, thereby pushing the particle formulation further within the SCS.

### Injection Into the Rabbit Eye Ex Vivo

Albino New Zealand White rabbit eyes (Pel-Freeze, Rogers, AR, USA) were used for ex vivo injection studies. Frozen rabbit eyes were thawed in a water bath at room temperature (∼22°C) for 30 minutes; unnecessary ocular tissues, such as muscles, conjunctiva, and fat, then were removed from the eyes. To generate an IOP of 10 to 15 mm Hg, 1× Hank's balanced salt solution (HBSS; Mediatech, Manassas, VA, USA) was injected into the vitreous, and the IOP was measured by a tonometer (iCare Tonovet, Helsinki, Finland). Then, SCS injection using a microneedle was performed across the sclera superonasally 3 mm posterior to the limbus. All SCS injections were accomplished using a 30-gauge hollow microneedle with 750 μm length that was kindly provided by Clearside Biomedical (Alpharetta, GA, USA).

We filled two formulations sequentially into a disposable syringe (1 mL Luer-lock plastic syringe; BD Bioscience, San Jose, CA, USA): a hydrogel formulation made of HA (Lifecore, Chaska, MN, USA) and a particle formulation containing carboxylate-modified red-fluorescent microspheres (2 μm diameter, FluoSphere, 580/605 nm; Life Technologies, Carlsbad, CA, USA). First, 30 μL 4% (wt/vol) HA (2.6 MDa) hydrogel was filled in the syringe as a drug-pushing material (the HA hydrogel formulation). Then, the syringe was filled with 20 μL of a model drug formulation, 0.5% (wt/vol) red-fluorescent microspheres in 1% (wt/vol) HA (the particle formulation). Due to the viscosity of the HA formulation, the two formulations did not mix in the syringe. A combined total 50 μL volume of both formulations was infused into the SCS in one injection. To minimize backflow of the formulations, the syringe was not withdrawn from the injection site until 1 minute injection.

While the standard formulations are discussed above, during initial studies, the concentration of HA in both formulations was varied. Also, silicone oil (500 and 30,000 cP, Sigma-Aldrich Corp., St. Louis, MO, USA) was used instead of HA solution as the pushing material in other studies, as indicated in the text.

### Incubation of the Rabbit Eye Ex Vivo

The ex vivo rabbit eyes that had been injected with the formulations were placed in the water bath containing HBSS buffer. The eyes were incubated at 37°C for 6 hours to induce swelling of the HA hydrogel in the SCS.

### Injection Into the Rabbit Eye In Vivo

In vivo injection was performed using Albino New Zealand White rabbits (Charles River Breeding Laboratories, Wilmington, MA, USA). The procedures of the animal study were approved by the Georgia Institute of Technology Institutional Animal Care and Use Committee, and practices complied with the ARVO Statement for the Use of Animals in Ophthalmic and Vision Research.

As a first step, rabbits received a subcutaneous injection for anesthesia, a mixture of ketamine HCl (17.5 mg/kg, Ketathesia; Henry Schein, Dublin, OH, USA) and xylazine (8.5 mg/kg, AnaSed Injection; Akorn, Lake Forest, IL, USA). To maintain anesthesia, isoflurane gas was administered to the rabbit until the SCS injection was finished. Three minutes before the injection, one or two drops of 0.5% proparacaine HCL (Bausch+Lomb, Bridgewater, NJ, USA) was applied to the eye for local anesthesia. The SCS injection, comprising a total of 50 μL containing the particle and HA hydrogel formulations, was administered across the sclera supranasally 3 mm posterior to the limbus. After injection, buprenorphine HCl (0.3 mg/mL; Par Pharmaceuticals, Spring Valley, NY, USA) was injected intramuscularly at a dose of 0.03 mg/kg to reduce pain. All rabbits were euthanized two days after injection with pentobarbital (150 mg/kg; SomnaSol, Henry Schein). The eyes were enucleated and splayed to analyze the particle distribution in the SCS.

### Analysis of Particle Delivery in the SCS

The rabbit eyes were immediately immersed in 100% isopropyl alcohol, then directly transferred into liquid nitrogen to preserve the post-SCS injection distribution of particles. The completely frozen eyes were cut by a razor from the optic nerve to the limbus to make eight symmetric petals. Then, the cut petals were peeled off and splayed like a flower to observe the particle distribution in the eye from the chorioretinal side. The ocular petals were imaged by a camera (Cannon 60d; Canon, Melville, NY, USA) to generate bright field and fluorescence images (λex = 580/ λem = 605). A green LED light (Bluewind Multicolor RGB; HitLight, Baton Rouge, LA, USA) was used as a light source and an optical bandpass filter (610 ± 10 nm; Edmunds Optics, Barrington, NJ, USA) was mounted on the camera to get the desired fluorescence images.

The SCS petals were divided and sliced according to the distance from the limbus (0–3, 3–6, 6–9, and >9 mm) to calculate the particle distribution. To extract the particles, the petals were lysed and sonicated in radioimmunoprecipitation assay buffer (Abcam, Cambridge, UK) to disrupt the tissues. Then, the fluorescent signals of the extracted-particle solutions were measured by a plate reader (Synergy Microplate Reader, Winooski, VT, USA).

### SCS Thickness Measurement

To measure SCS thickness of the rabbit eye in vivo, an ultrasound B scanner (UBM Plus, Accutome, Malvern, PA, USA) was used. The thickness was measured right after the injection, 4 hours later, and 2 days later in the eight positions of the ocular globe (Fig. 8a). At least three in vivo eyes were used to measure the thickness change as a function of time and ocular location.

### IOP Measurement

To determine IOP changes after SCS injection, IOP of the rabbit eye in vivo was measured daily for 1 week using a rebound tonometer (from 10 AM to 12 PM). However, the IOP was not measured on the day of injection due to effect of ketamine anesthetic on IOP.^48^ IOP measurements were averaged from at least five measurements. The baseline IOP was determined by measuring IOP every day for 1 week before injection.

### Statistical Analysis

At least three replicates were obtained for each data set, and the mean and standard deviation were calculated. To assess statistical significance, 1-way ANOVA with replicates was used for data analysis, and *P* values < 0.05 were considered significant.

## Results

### Formulation Optimization

**Figure 2:**
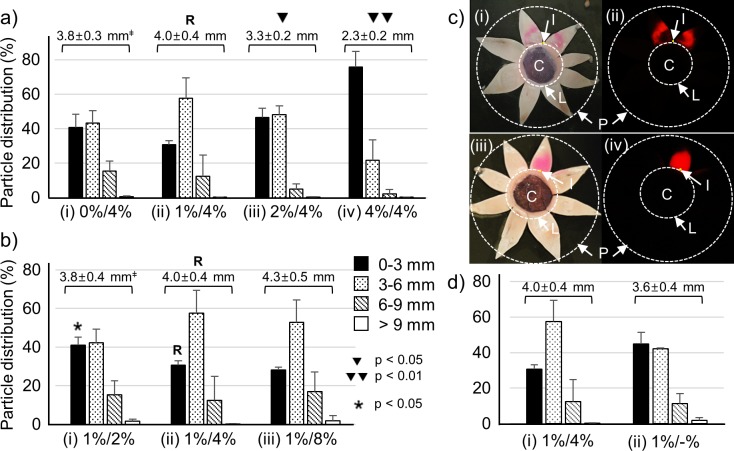
Optimization of HA concentration in the particle and hydrogel formulations in a syringe. (a) Distributions of red-fluorescent particles in the SCS with different HA concentrations in the particle formulation after injection into the SCS of ex vivo rabbit eyes: (i) 0%, (ii) 1%, (iii) 2%, (iv) 4% HA. The HA concentration of the pushing hydrogel was fixed to 4%. (b) Distributions of red-fluorescent particles in the SCS with different HA concentrations in the pushing hydrogel: (i) 2%, (ii) 4%, (iii) 8%. The HA concentration in the particle formulation was fixed to 1%. (c) Representative bright field (i, iii) and fluorescence (ii, iv) images subsequent to SCS injection of 1% HA in the particle formulation and 4% HA in the hydrogel formulation (upper images) and 1% HA in the particle formulation with no hydrogel formulation (bottom images). (d) Distributions of red-fluorescent particles in the SCS: (i) 1% HA in the particle formulation and 4% HA in the hydrogel formulation and (ii) 1% HA in the particle formulation with no hydrogel formulation. ‡Average particle distance (APD) (mm) = (1.5 mm × the particle distribution % of 0–3 mm area) + (4.5 mm × the particle distribution % of 3–6 mm area) + (7.5 mm × the particle distribution % of 6–9 mm area) + (10.5 mm × the particle distribution % of 9 mm area). R is reference value. ▾, ▾▾ indicate significance of differences (1-way ANOVA, P < 0.05) between the APT of the reference (R) and the APT of the formulations. *Significance of differences (1-way ANOVA, P < 0.05) between the 0–3 mm distribution of the reference (R) and other formulations. Graphs (a and b) present mean ± SD based on three replicate samples. I, injection site; C, cornea; L, limbus; and P, posterior pole.

We optimized the particle and hydrogel formulations for the best posterior delivery by controlling HA concentration in the formulations. First, HA concentration in the particle formulation was varied between 0%, 1%, 2%, and 4% (wt/vol), and HA concentration in the hydrogel formulation was fixed at 4%. Each formulation was injected into the SCS of the rabbit eye ex vivo, and the particle distribution was analyzed to determine distance from the limbus, which was divided into four quadrants: 0–3, 3–6, 6–9, and >9 mm from the limbus (Fig. 2a).

The first aim of this study was to push the particle formulation as far to the back of the SCS as possible, without mixing with the hydrogel formulation. Among the four formulations in Figure 2a, the 1%/4% (particle formulation/hydrogel formulation, Fig. 2a[ii]) pushed more particles to the back (70% of particles were more than 3 mm from the limbus) than the others. As the viscosity difference between the particle and hydrogel formulations decreased, the pushing effect also decreased (i.e., 2%/4% and 4%/4% formulation in Figs. 2a[iii] and 2a[iv]) probably because of increased resistance to flow due to higher viscosity.

Interestingly, when the particle formulation had no HA (0% HA, Fig. 2a[i]), the efficiency (59% more than 3 mm from the limbus) was less than that of the 1% HA formulation. This could be because the low-viscosity (0% HA) particle formulation can spread well, possibly around the pushing hydrogel into the space between the hydrogel formulation and SCS wall. In addition, the 0% HA particle formulation can be mixed with the hydrogel formulation more easily than the others, because there is less interaction between the solvent and particles. With just 1% HA, the viscosity is larger and the particles can interact with the long-chain polymers when they move.^27,42^

To describe the particle pushing in another way, the average particle distance (APD) from the limbus was calculated based on the particle distribution and is shown above each of the distribution profiles in Figure 2a. The APD of the 1%/4% (R, reference; Fig. 2a[ii]) had the largest value of 4.0 mm, which was significantly greater than the APD of the 2%/4% (Fig. 2a[iii]) and 4%/4% (Fig. 2a[iv]) formulations. In summary, 1% HA in the particle formulation was found to be best.

Next, the concentration in the hydrogel formulation was varied between 2%, 4%, and 8%, and the HA concentration of the particle formulation was fixed to 1% (Fig. 2b). Consistent with our expectation that larger viscosity differences usually are helpful, the 2% HA hydrogel formulation only pushed 59% of the particles more than 3 mm from the limbus, whereas the 4% and 8% HA hydrogel formulations pushed 70 and 72% of the particle past the 3 mm mark, respectively. This pushing efficiency, as well as the APD (4.3 mm) of the 8% HA hydrogel were the largest among the formulations considered, but were not significantly different from the 4% HA hydrogel. In addition, the 8% HA hydrogel entrapped bubbles that were not easily removed, due to the high viscosity. Thus, we chose the 4% HA formulation as the pushing hydrogel in combination with the 1% HA particle formulation for further study.

To better understand particle distribution in the SCS, the eye was dissected and imaged after injection using the 1%/4% HA formulation (Fig. 2c). In the bright field image (Fig. 2c[i]), the red particles in 1% HA were pushed without mixing by the 4% HA hydrogel from the site of injection. The fluorescence image (Fig. 2c[ii]) also shows the discrete areas of the particle and hydrogel formulations. Note that while the fluorescence image may appear to show particles pushed to the side rather than toward the back of the SCS, this is an artifact of the flat-mount presentation of what is a spherical tissue. Comparison with the bright field image shows that the pushing actually was toward the posterior SCS due to the way the eye was sectioned. To further assess the effect of the pushing hydrogel formulation, particles in 1% HA without the hydrogel formulation (1%/-%) were injected into the SCS. The bright field and fluorescence images (Fig. 2c[iii], 2c[iv]) show that the particle formulation was localized near the injection site without pushing and further spreading.

The particle distribution shows the particle pushing effect by the hydrogel formulation quantitatively (Fig. 2d). While the 1%/4% formulation pushed 70% particles more than 3 mm from the limbus (Fig. 2d[i]), the 1%/-% formulation delivered only 55% particles more than 3 mm from the limbus (Fig. 2d[ii]). The APD of the 1%/-% formulation (3.6 mm) also was less than that of the 1%/4% formulation (4.0 mm).

### Effect of Body Temperature on Hydrogel Swelling

In addition to pushing the particle formulation by the hydrogel at the time of injection (i.e., studied above), our next aim was to study swelling of the HA hydrogel in the SCS to further spread the particles to the back of the eye. We hypothesized that a temperature increase from room to body temperature would reduce the viscosity of the hydrogel, leading the hydrogel in the SCS to spread toward the back of the eye, thereby pushing the particles.^36–38^ To achieve this aim, rabbit eyes were injected with pushing formulations ex vivo and then incubated at different temperatures: 4°C, room temperature, and 37°C for 6 hours. Incubation at 37°C was intended to simulate in vivo injection at body temperature. Since the clearance time of commercial HA hydrogel (Discovisc; Alcon Laboratories, Duluth, GA, USA) from SCS was reported as 6 hours, we selected a 6-hour incubation time.^49^

At first, we checked the robustness of the SCS after 6 hours incubation at 37°C and found that the eyes did not lose their IOP, and remained firm to the touch. To confirm whether the SCS had been damaged or if leakage of the formulation had occurred, the rabbit eye was frozen and dissected (Supplementary Fig. S1). In the cross-sectional image, the formulation was seen to remain in the SCS without leakage to the vitreous humor and the red particles were seen to have been pushed by the hydrogel.

**Figure 3:**
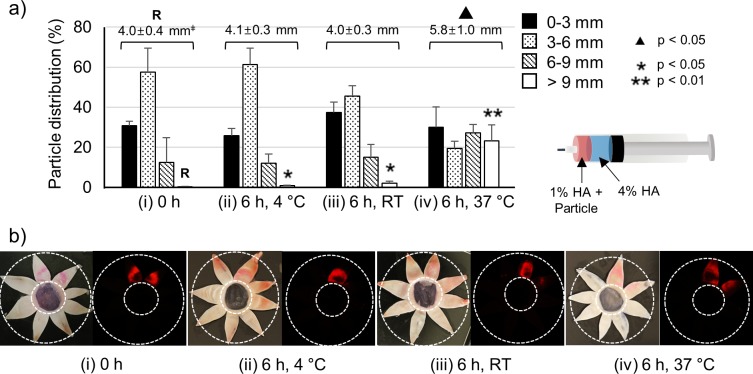
Analysis of particle delivery toward the back of the eye by hydrogel pushing as a function of incubation temperature and time. The optimized formulation of 1% HA in the particle formulation and 4% HA in the hydrogel formulation was injected into the SCS of ex vivo rabbit eyes. (a) Distributions of red-fluorescent particles in the SCS at (i) 0 hours, (ii) 6 hours at 4°C, (iii) 6 hours at RT, and (iv) 6 hours at 37°C after SCS injection. (b) Representative bright field (left) and fluorescence images (right) of red-fluorescent particles in the SCS at (i) 0 hours, (ii) 6 hours at 4°C, (iii) 6 hours at RT, and (iv) 6 hours at 37°C after SCS injection. The inner and the outer white dashed lines indicate the boundary of the limbus and the optic nerve, respectively. ‡APD (mm). R is reference value. ▴ indicates significant difference (1-way ANOVA, P < 0.05) between the APD of the reference (R) and the APD of the formulations. *, **Significant difference (2-way ANOVA, P < 0.05) between the 9-mm distribution of the reference (R) and other formulations. Graphs (a) present mean ± SD based on three replicate samples.

To observe the effect of incubation temperature on hydrogel swelling, ex vivo rabbit eyes injected with the formulation were incubated for 6 hours at different temperatures and the particle distribution and APD value were calculated (Fig. 3a). The 4°C incubation (Fig. 3a[ii]) did not change particle distribution or delivery to the back of the SCS compared to the preincubation observation (Fig. 3a[i]). After room-temperature incubation (Fig. 3a[iii]), the particle distribution was not significantly changed, although there may have been some spreading toward the back and toward the front of the SCS. In contrast, eyes incubated at 37°C increased particle delivery to the posterior SCS (Fig. 3a[iv]). The percentage of particles located more than 6 mm from the limbus increased 4-fold from 13% (no incubation) to 51%. Specifically, the percentage of particles beyond the 9-mm threshold dramatically increased, from 0.3% to 23% after the 37°C incubation. This indicated that hydrogel pushing during injection can help move particles posteriorly in the SCS, but postinjection hydrogel swelling can have an even greater effect.

These findings are supported by bright field and fluorescence microscopy of the particle distributions (Fig. 3b). Before incubation, the particles were separate from the hydrogel and located toward in the anterior SCS. After incubation at 4°C and room temperature, particle distributions were similar to before incubation. Particles generally did not reach to the outer white dashed line, which identified the location of the optic nerve in Figures 3b(ii) and 3b(iii). Particles in the SCS of eyes incubated at 37°C were able to reach the optic nerve, and the area of particle spread was increased toward the back of the eye due to HA hydrogel pushing.

**Figure 4:**
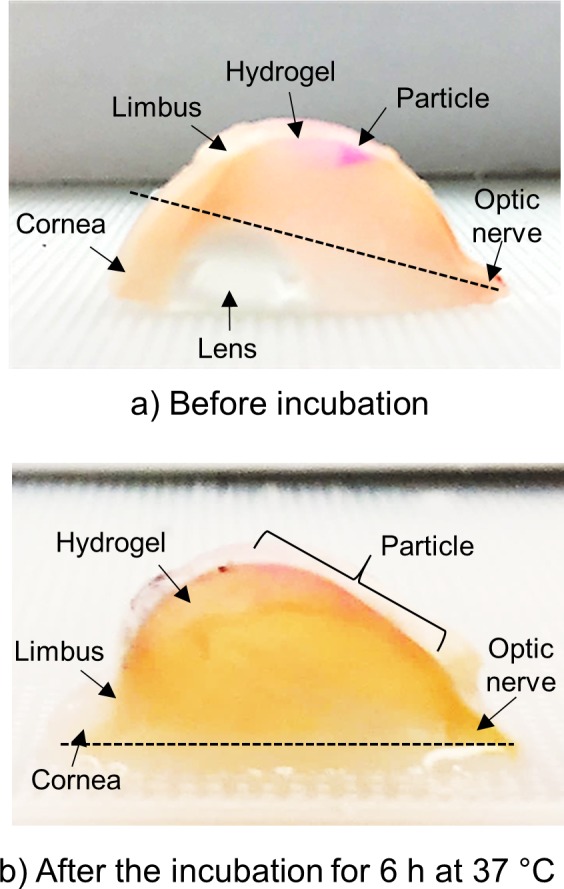
Representative photographic images of a dissected eye right after SCS injection (a) and 6 hours after injection while incubated at 37°C (b) of red-fluorescent particles into the SCS of the ex vivo rabbit eye. The dashed line indicates the same location of the eye in both images to facilitate comparison.

To further study the extent of particle pushing and spreading, the eyes were dissected immediately after injection (Fig. 4a) and after 6 hours of incubation at 37°C (Fig. 4b). Before incubation, the red particles were pushed by the hydrogel formulation and located only part-way back in the SCS (Fig. 4a). After incubation, the particles were spread toward the optic nerve by hydrogel pushing and swelling (Fig. 4b).

**Figure 5:**
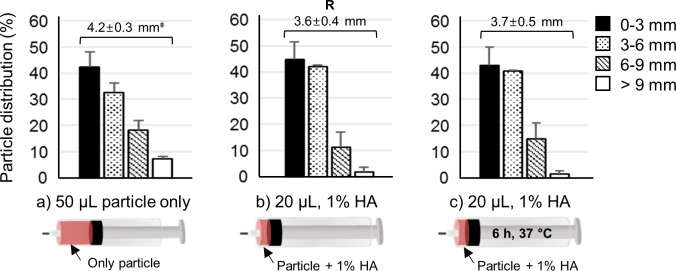
SCS injections into the ex vivo rabbit eye without a pushing hydrogel formulation. (a) Distributions of red-fluorescent particles in the SCS with (a) 50 μL particles in HBSS buffer, (b) 20 μL particles in 1% HA, and (c) 20 μL particles in 1% HA with incubation for 6 hours at 37°C after SCS injection. ‡APD (mm). R is reference value. No significant differences were found (1-way ANOVA, P > 0.05) between the APD of the reference (R) and the APD of the other formulations. All graphs present mean ± SD based on three replicate samples.

Until now, all injected formulations contained a hydrogel pushing formulation. Therefore, we tested additional negative control formulations to better understand the role of hydrogel pushing. First, 50 μL particle solution without HA was injected into the SCS of the rabbit eye ex vivo (Supplementary Fig. S2a). Only 7% of the particles were delivered to the most posterior region of SCS (> 9 mm from the limbus), and the APD value was 4.2 ± 0.3 mm (Fig. 5a). We next injected 20 μL of a particle formulation in 1% HA without a companion pushing hydrogel formulation (Fig. 5b), and the eyes were incubated at 37°C for 6 hours (Fig. 5c). After incubation, the particle distributions between the particles in 1% HA with and without incubation at 37°C were similar; the APD also was almost the same (3.6 and 3.7 mm, respectively). Images of the particle distribution (Supplementary Figs. S2b, S2c) also were similar, revealing that the particle formulation did not swell or spread during the incubation in the absence of a pushing hydrogel formulation. These data further supported the mechanism that particle delivery to the posterior SCS was caused largely by hydrogel swelling and spreading at elevated temperature.

### Analysis of Other Pushing Materials

**Figure 6:**
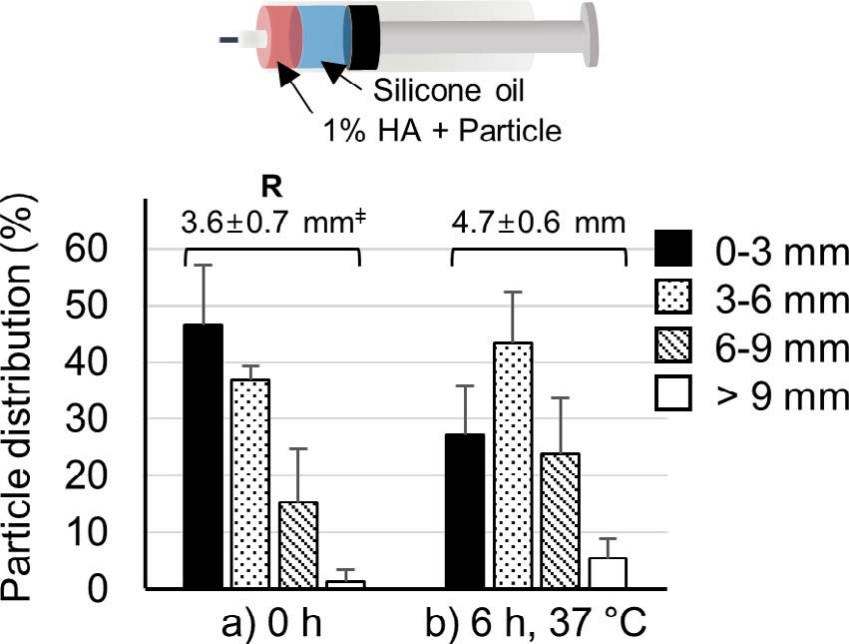
Analysis of particle delivery toward the back of the eye by silicone oil pushing. Distributions of red-fluorescent particles in the SCS (a) immediately after injection (0 hours) and (b) 6 hours after injection, incubated at 37°C. ‡APD (mm). R is reference value. There was no statistical significance (1-way ANOVA, P > 0.05) between the APD before incubation and after incubation. Graphs present mean ± SD based on three replicate samples.

To further understand the role of hydrogel swelling, we used silicone oil instead of HA hydrogel as the pushing formulation. Silicone oil is viscous and immiscible with the aqueous particle formulation. Moreover, silicone oil is used in ocular surgery, indicating its safety for injection into the SCS.^50^ After SCS injection with the silicone oil formulation, we quantified particle distribution (Fig. 6), and created images of the dissected tissue “petals” immediately following injection (Supplementary Fig. S3a) and after incubation at 37°C for 6 hours (Supplementary Fig. S3b).

The particle formulation and silicone oil remained separate, but pushing was less effective compared to the HA hydrogel formulation (Supplementary Fig. S3a). The particle formulation was not delivered effectively toward the back of the eye, and instead migrated to the side and front of the SCS (Fig. 6a). After incubation, the particle formulation appeared to have moved toward the back of the SCS (Fig. 6b), possibly because of reduced viscosity of silicone oil at elevated temperature, which led to spreading, but the differences were not statistically significant. Moreover, the particle formulation appeared to form an emulsion in the silicone oil rather than being pushed away (Supplementary Fig. S3b). This indicated that the pushing material should be hydrophilic as well as difficult to mix (in part due to viscosity differences) with the drug particle formulation.

### Enhancement of Hydrogel Pushing Using Osmotic Flow

In the best result so far (Fig. 3a[iv]), approximately 50% of the particle formulation was delivered to the posterior SCS (>6 mm from the limbus) after incubation at 37°C for 6 hours. To further increase pushing to the posterior SCS, we added a high salt concentration to the HA hydrogel formulation to create a hyperosmotic environment leading to increased osmotic flow into the hydrogel and thereby greater swelling and pushing.^51,52^

**Figure 7:**
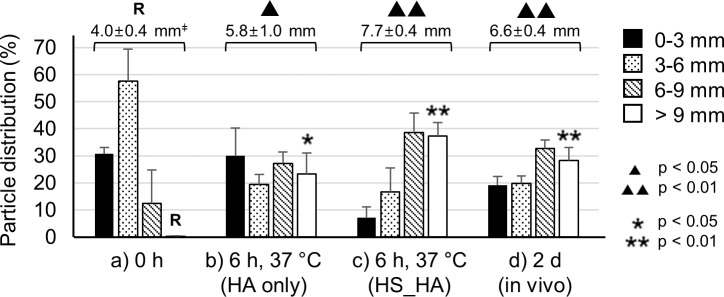
Analysis of particle delivery toward back of the eye by a high-salt HA (HS_HA; 9% NaCl) hydrogel formulation. Distributions of red-fluorescent particles in the SCS (a) right after injection (0 hours), (b) 6 hours after incubation at 37°C with the HA hydrogel, (c) 6 hours after incubation at 37°C with the HS_HA hydrogel, and (d) 2 days after in vivo injection using the HS_HA hydrogel formulation. ‡APD (mm). R is reference value. ▴, ▴▴ indicates significant difference (1-way ANOVA, P < 0.05) between the APD of the reference (R) and each incubation condition. *, **Significant difference (1-way ANOVA, P < 0.05) between the >9 mm distribution of the reference (R) and other formulations. Graphs present mean ± SD based on three replicate samples.

When comparing the particle distribution with the high-salt hydrogel formulation (Fig. 7c) to the “conventional” formulation without high-salt, the percentage of particles delivered to the posterior SCS (>6 mm from the limbus) was significantly increased, from 50% to 76% (Figs. 7b, 7c). Moreover, the distribution to the most posterior area (> 9 mm) increased to 37% of particles (Figs. 7a, 7c). Also, there was a statistically significant difference between the distribution of particles further than 9 mm between the HA hydrogel and the high-salt HA hydrogel (Figs. 7b, 7c). The APD value of the high-salt HA was drastically increased, to double the APD from before incubation. The normal HA hydrogel (Fig. 7b) increased the APD to 5.8 mm after incubation; the high-salt HA hydrogel (Fig. 7c) increased the APD to 7.7 mm. Thus, we concluded that the high-salt hydrogel formulation dramatically enhanced particle delivery to the back of the eye.

### In Vivo Particle Delivery by Hydrogel Pushing

Encouraged by these findings in the rabbit eye ex vivo, we performed SCS injection into the New Zealand white rabbit eye in vivo using the high-salt HA hydrogel formulation. Upon euthanasia two days later, the eyes were enucleated and frozen for dissection. The particle distribution in the in vivo rabbit eyes is shown in Figure 7d, based on the petal image in Supplementary Figure S4b. Overall, particle distribution was shifted toward the posterior SCS two days after injection in vivo, but to a somewhat lesser extent compared to the ex vivo findings after 6 hours of incubation.

More specifically, particle distribution to the posterior SCS (>6 mm from the limbus) was not significantly different in the ex vivo eyes after the 6-hour incubation (76%) from the in vivo eyes (61%). The APD value (7.7 mm) in the ex vivo eyes incubated for 6 hours was significantly greater than that in the in vivo eyes (6.6 mm; Fig. 7). The differences between particle distributions in the ex vivo eye after 6-hour incubation compared to the in vivo eye 2 days after injection may be explained by the rabbits' movement and other differences in conditions between in vivo and ex vivo, as well as the different timing of analysis (6 hours ex vivo versus 2 days in vivo).

**Figure 8:**
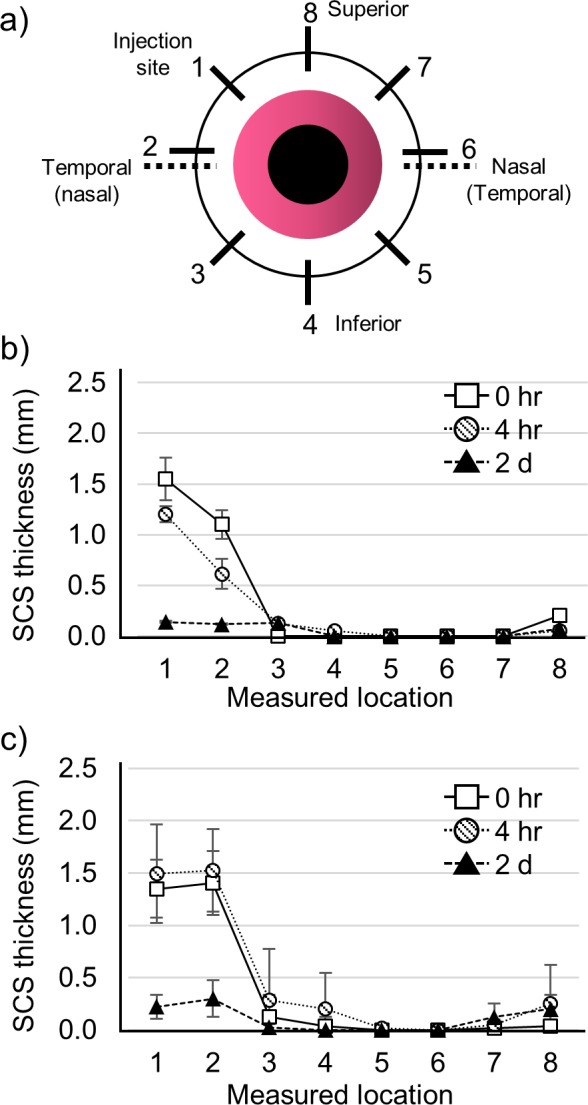
Schematic illustration of the measurement locations on the rabbit eye in vivo (a). Measurement of SCS thickness change due to hydrogel spreading after 2 days with the conventional HA hydrogel (b) and the high-salt HA hydrogel (c) formulation. Graphs present average ± SD based on three replicate samples.

We hypothesized that high-salt hydrogel formulation increases pushing by increasing osmotic water flow into the SCS. To assess this hypothesis, SCS thickness of the rabbit eye in vivo was measured by ultrasound B scan. Thickness was measured right after injection (0 hours), as well as 4 hours and 2 days after injection at eight locations around the ocular globe (Fig. 8a) after injection with conventional HA hydrogel formulation (Fig. 8b) and high-salt HA hydrogel formulation (Fig. 8c).

After injection of the conventional HA hydrogel, SCS thickness peaked at 1.4 mm and then declined until it was almost cleared after 2 days (Fig. 8b). Conversely, SCS thickness after injection with the high-salt hydrogel formulation peaked at 1.8 mm over a larger region of the SCS compared to the conventional HA hydrogel. This thickness increase supports the interpretation that influx of water due to osmotic flow into the SCS was enhanced by the use of the high-salt hydrogel formulation. We concluded that osmotic flow generated by the high-salt hydrogel enhanced the posterior particle delivery by HA hydrogel swelling.

### Effect of Hydrogel Injection Into SCS on IOP Change

IOP change was measured to determine the effect of hydrogel injection into the SCS. After injection of particles and pushing hydrogel, IOP decreased to approximately 4.0 mm Hg and then gradually recovered to the normal IOP (10–11 mm Hg) at approximately 6 days after injection (Supplementary Fig. S5). Reduced IOP could be due to multiple factors, including inflammatory response to the SCS injection, decreased aqueous humor secretion due to disruption of ciliary body function, and/or increased uveoscleral outflow due to stretching of the SCS by the hydrogel that also may expand the trabecular meshwork through increased tension on the scleral spur.^24,53,54^

## Discussion

Drug delivery localized to the eye, for example by injection, increases ocular bioavailability while reducing systemic exposure compared to systemic drug administration. However, targeting drug delivery within the eye can provide still greater specificity to a drug's site of action while reducing off-target effects at other locations. Injection into the SCS targets drug delivery to the chorioretina with reduced drug exposure to the anterior segment or dilution in vitreous humor.^2,9–15^ However, many indications are located in specific regions of the posterior segment, such as the macula. Through the combination of SCS injection and swollen hydrogel pushing, this study demonstrated the ability to focus drug delivery specifically to the posterior SCS near the macula and optic nerve. Such targeting could enhance therapies of posterior segment indications located at the back of the eye.

The particle delivery in this system can be divided in two distinct mechanisms: pushing during the injection and swelling after the injection. By the first mechanism, the particle formulation can be pushed by the pressure from the HA hydrogel formulation. To accomplish this, the hydrogel should be immiscible, hydrophilic, and more viscous than the particle formulation. The formulations that had a higher viscosity difference between the particle and hydrogel formulations showed a better pushing effect in the formulation screening.

The HA hydrogel is physically synthesized by hydrogen bonding among the HA molecules.^28,32^ As the concentration of the HA hydrogel increases, more hydrogen bonds are formed. In addition, viscosity and mechanical strength increase together as the concentration increases.^42–45^ Thus, highly concentrated hydrogel is more advantageous for use as a pushing material. In contrast, the particle formulation should ideally have lower viscosity than the hydrogel formulation so that the pushing strength from the hydrogel will more effectively move the particles. Thus, a low concentration of HA is more advantageous for the particle formulation.

By the second mechanism, the postinjection hydrogel swelling in the SCS transferred the particles further toward the back of the eye. The swelling occurred not only as the result of temperature increase but also due to osmotic flow of water into the hydrogel.^27,31,39,45,46^ Indeed, since viscosity and mechanical strength of the hydrogel decrease as the temperature increases, the hydrogel injected into the eye can swell and spread well toward the posterior SCS.^40,41^ The use of a high-salt HA hydrogel formulation enhanced posterior delivery more than the use of the conventional HA hydrogel formulation (Fig. 7). The high salt concentration in the hydrogel formed a hyperosmotic condition in the SCS that drove osmotic flow into the SCS from the adjacent tissues.^45,46^

While hydrogel pushing is desirable, it is specifically intended to push posteriorly, as opposed to anteriorly or to the side. Because injections were performed near the limbus, eye anatomy provides a physical barrier that prevents significant anterior pushing. While we do not have reason to believe that hydrogel pushing is otherwise directional, it is useful to recognize that the eye is spherical, so that the flat-mount tissue images of particle distribution shown in this study can be deceiving. Because of the spherical geometry, flow from a site near the limbus preferentially goes posteriorly because that is where there is more space to flow. As the formulation reaches the equator, the situation reverses, such that flow to the side is preferred to posterior flow due to geometry.^55^ However, once the formulation has passed the equator, flow to the side often may be advantageous to spread across the most posterior chorioretina, including the macula.

However, there are limitations to targeting delivery to the posterior segment of the eye by swollen hydrogel pushing. First, the delivered amount of drug was reduced due to the hydrogel volume occupying 60% in the formulation (20 μL drug formulation in total injection volume of 50 μL), thereby possibly reducing the maximum dose administered. Second, the significance of the IOP reduction after SCS injection should be investigated further, because there can be safety concerns associated with excessively low IOP.^56,57^

In this study, we selected HA hydrogel as the material to push the particles to the back of the eye. HA was used because it is a component of the vitreous humor and, thus, is safe and biodegradable in the body, as shown in many ophthalmic and other products.^29–31^ Physically synthesized HA hydrogel was used not only to facilitate swelling and spreading, but for rapid clearance from the body. Since the hydrogel appeared to be cleared within a few days after the injection (i.e., as shown be collapse of the SCS to its original thickness in Fig. 8 and return to normal IOP in Supplementary Fig. S5), repeated injections into the SCS using pushing hydrogels should not be inhibited by residual hydrogel if the treatments are infrequent (e.g., monthly). The polymeric microsphere particles were used as a drug model to simulate a sustained-release, particulate drug formulation. Moreover, since particle delivery relied on hydrogel pushing and swelling, the particles did not need custom modification or engineering to be moved in the SCS. We believe that this system is well suited to drug delivery targeting the macula, optic nerve, or other posterior chorioretinal targets. To our knowledge, this is the first study to use a hydrogel to push material in the eye for posterior segment delivery. These initial findings prompt the need for more studies and additional optimization to more fully assess the strengths and weaknesses of targeted delivery in the SCS by swollen hydrogel pushing.

## Conclusions

The objective of this project was to deliver model drug particles to the back of the eye using hydrogel swelling and pushing. We accomplished this using a particle formulation containing 1% HA and a hydrogel formulation containing 4% HA, which was physically synthesized by hydrogen bonding, that were introduced sequentially from a single syringe. The formulations did not mix in the syringe during short-term storage. Upon injection via microneedle into the SCS of the rabbit eye ex vivo and in vivo, the particle formulation was infused first, followed by the hydrogel formulation. Due to the viscoelastic property of the hydrogel, the particle formulation was pushed posteriorly toward the middle of the SCS by the hydrogel. After the injection, the hydrogel swelled and spread further toward the posterior SCS, pushing particles to the macula and optic nerve. Increasing temperature of the eye from room temperature to 37°C, or injecting in vivo, weakened the hydrogen bonds in the hydrogel, causing a decrease of viscosity and mechanical strength of the hydrogel, thereby allowing it to swell and flow in the SCS.

Osmotic flow into the SCS and hydration of the hydrogel also enhanced hydrogel swelling by diluting it. To further increase hydrogel swelling, a high salt concentration (9% NaCl) was added to the HA hydrogel formulation. Since the high-salt HA hydrogel generated a hyperosmotic condition in the SCS, the osmotic flow and hydration effect were increased. In this way, up to 76% of particles were delivered to the posterior SCS (>6 mm from the limbus).

In this system, the micrometer-size particles were delivered by bulk flow caused by hydrogel pushing without requiring modification or functionalization of the particles or administering external driving forces. Furthermore, diverse drugs that can be prepared in particle form could be used for delivery. The hydrogel, a 4% HA solution, is biodegradable and safe, and will be degraded rapidly in the eye. We concluded that ocular drug delivery using swollen HA hydrogel pushing is a promising method for targeting drug delivery to the back of the eye.

## Supplementary Material

Supplement 1
