# Supplementary material for: Targeted Drug Delivery in the Suprachoroidal Space by Swollen Hydrogel Pushing
Source: Invest Ophthalmol Vis Sci. 2018 Apr;59(5):2069–79. doi: 10.1167/iovs.17-23758 (PMC5909801; doi:10.1167/iovs.17-23758)
Supplement: Supplement 1 [file iovs-59-05-14_s01.pdf]

**Supplementary Information**

# Targeted Drug Delivery in the Suprachoroidal Space by Swollen Hydrogel Pushing

*Jae Hwan Jung, Patcharin Desit, and Mark R. Prausnitz*

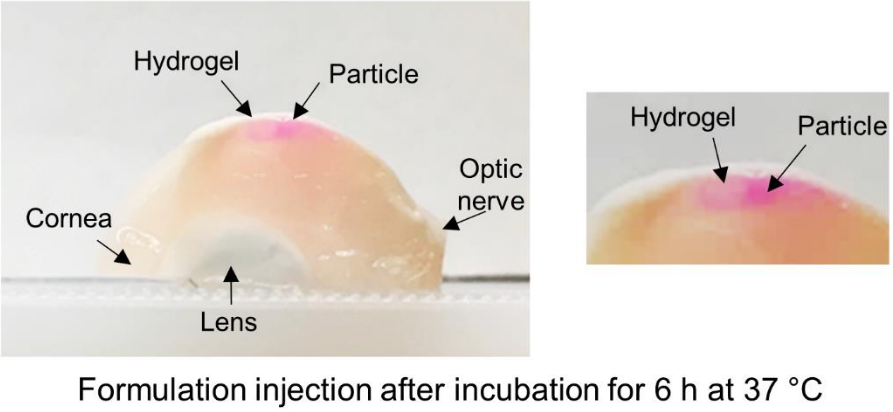


**Figure S1.** Representative image of a dissected eye after SCS injection of the 1% HA / 4% HA formulation in the rabbit eye ex vivo. To assess the role of possible degradation of the eye tissue, the eye was incubated for 6 h at 37 °C before the injection.

## Calculating specific viscosity of physically synthesized hyaluronic acid (HA) hydrogel

The specific viscosity ($\eta_{sp}$) of an HA solution is related to the intrinsic viscosity (η) and HA concentration (c)[1-3]. This relationship is shown as

$$\eta_{sp} = c[\eta]\left(1 + k'c[\eta] + \frac{(k'c[\eta])^2}{2!} + \frac{(k'c[\eta])^3}{3!}\right) \qquad k' = 0.4 \qquad (1)$$

The intrinsic viscosity of HA is related to molecular weight (M)[1, 3].

$$[\eta] = 0.029\ M^{0.80} \qquad (2)$$

Since the molecular weight of HA used in this study is $2.6 \times 10^6$ Da, the intrinsic viscosity (η) is 3800 $cm^3$/g. Then, we can estimate specific viscosities ($\eta_{sp}$) of the hydrogels with different HA concentrations (c) based on the Equation (1) as.

| HA concentration | c, μg/$cm^3$ | c[$\eta$] | $\eta_{sp}$ |
|---|---|---|---|
| 1% | $1 \times 10^4$ | 38 | $3.1 \times 10^4$ |
| 2% | $2 \times 10^4$ | 76 | $4.5 \times 10^5$ |
| 4% | $4 \times 10^4$ | 152 | $6.9 \times 10^6$ |
| 8% | $8 \times 10^4$ | 304 | $1.1 \times 10^8$ |

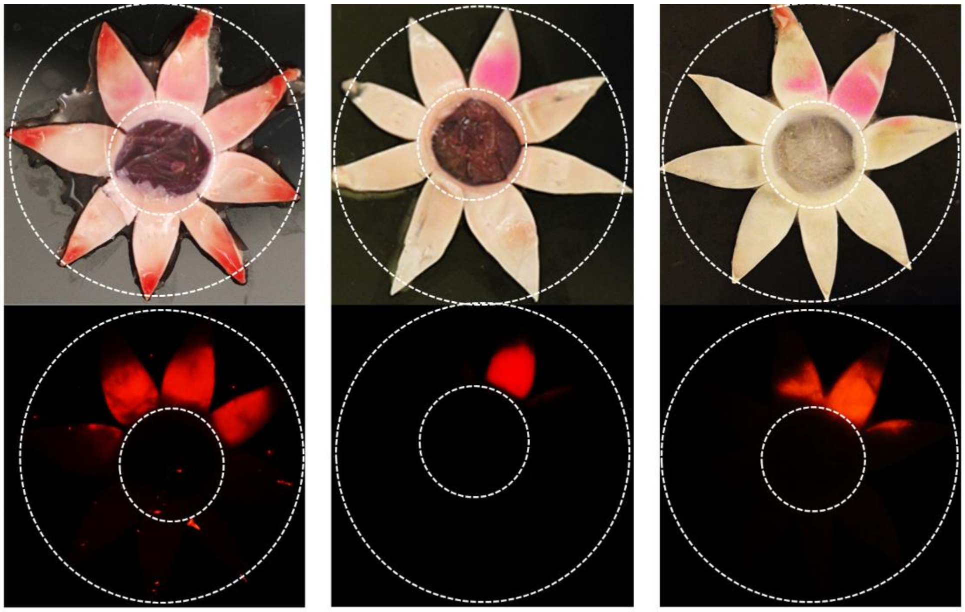


**Figure S2.** Representative bright field (top) and fluorescence images (bottom) of dissected rabbit eye petals after SCS injection of (a) 50 μL particles in water, (b) 20 μL particles in 1% HA, and (c) 20 μL particles in 1% HA followed by incubation for 6 h at 37 °C ex vivo.

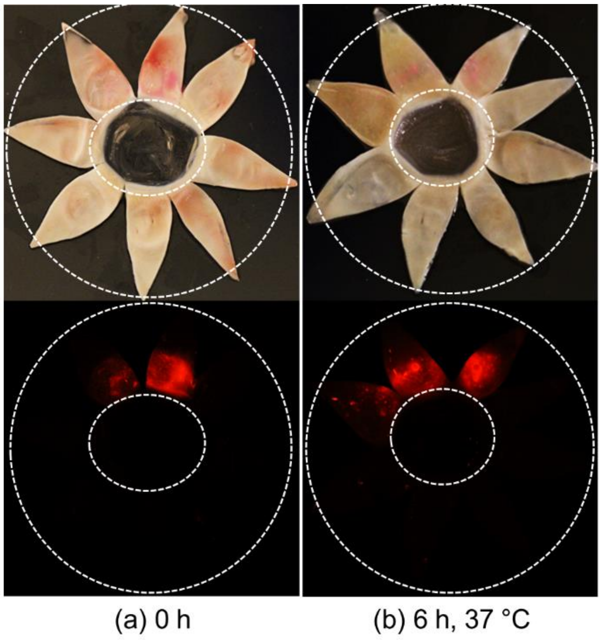


**Figure S3.** Representative bright field (top) and fluorescence images (bottom) of dissected rabbit eye petals (a) right after SCS injection (0 h) of a formulation consisting of particles and silicone oil and (b) after subsequent incubation at 37 °C for 6 h ex vivo.

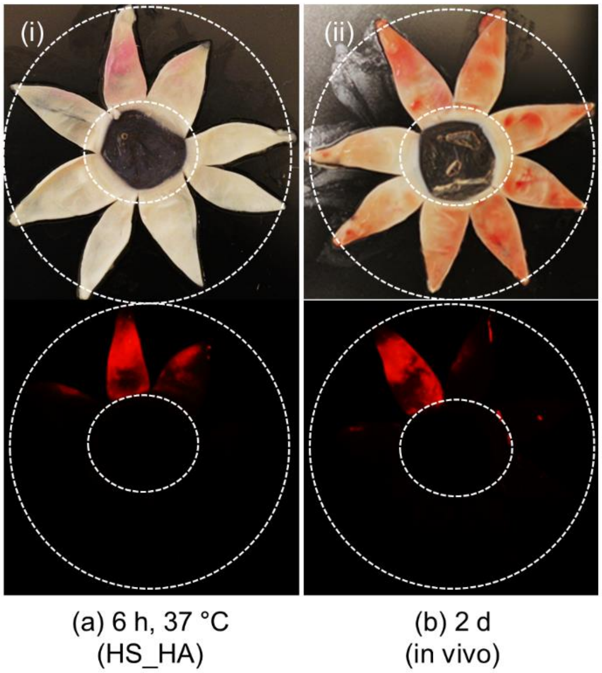


**Figure S4.** Representative bright field (top) and fluorescence images (bottom) of dissected rabbit eye petals after SCS injection (a) followed by 6 h incubation at 37 °C ex vivo and (b) followed by 2 days in vivo. The high-salt HA hydrogel formulation was used.

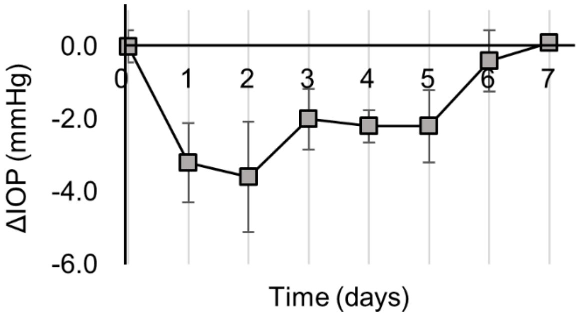


**Figure S5.** Effect of HA hydrogel injection on IOP in the rabbit eye in vivo. Change in IOP from baseline over time after SCS injection of particles and HA hydrogel. Data are expressed as mean ± SD (n=5).

## References

1. Cowman MK, Schmidt TA, Raghavan P, Stecco A. Viscoelastic Properties of Hyaluronan in Physiological Conditions. *F1000Res* 2015;4:622.

2. Berriaud N, Milas M, Rinaudo M. Rheological study on mixtures of different molecular weight hyaluronates. *Int J Biol Macromol* 1994;16:137-142.

3. Cowman MK, Matsuoka S. Experimental approaches to hyaluronan structure. *Carbohydr Res* 2005;340:791-809.
